# Supplementary material for: Miniature neurotransmission is required to maintain Drosophila synaptic structures during ageing
Source: Nat Commun. 2021 Jul 20;12:4399. doi: 10.1038/s41467-021-24490-1 (PMC8292383; doi:10.1038/s41467-021-24490-1)
Supplement: Supplementary file 1 — Supplementary Information [file 41467_2021_24490_MOESM1_ESM.pdf]

Supplementary Figures and Tables

Banerjee et al. 2021

Supplementary Fig. 1. Additional muscle and synapse morphological features during ageing at conventional (25°C) culture temperatures.

a, Representative tonic (HB9>Gal4, UAS>GFP red) and phasic (VMAT=vesicular monamine transporter, green) terminals innervating A2 mvim muscles at day 3. $n = 4$ biologically independent samples.

b, A2 mvim muscle surface area remains unchanged during ageing.

c, Presynaptic terminal area declines during ageing.

d, Quantification of the total number of boutons during ageing.

e, Representative images of presence of presynaptic and postsynaptic proteins at day 5 (upper panel) and in fragmented boutons at day 75 (lower panel). Magnification is identical for all images. $n \geq 5$ biologically independent samples.

f, Representative presynaptic nerve terminals innervating proximal femur leg muscles at day 5 and day 75. Boutons are labelled with the neuronal membrane marker (red, mcherry), active zones are labelled with Brp (green) and muscles are labelled with phalloidin (blue). $n = 3$ biologically independent samples.

One-way ANOVA, $p = 0.14$, $R^2 = 0.05$, $n \geq 16$ samples per timepoint (b), ***$p<0.001$, $R^2 = 0.13$, $n \geq 27$ presynaptic terminals per timepoint (c), ***$p<0.001$, $R^2 = 0.52$, $n \geq 29$ presynaptic terminals per timepoint (d), followed by Tukey's multiple comparison tests were used to compare the mean of each time point with the mean of all other time points. Statistical analysis details with a precise value of '*n*' are reported in Supplementary Table 2 and in Source Data file. Scale bars, 40μm (a, upper panel), 10μm (a, bottom panel), 2μm (e and f). Data are represented as mean ± SD, n = biologically independent samples.

Supplementary Fig. 1. Additional muscle and synapse morphological features during ageing at conventional (25ºC) culture temperatures.

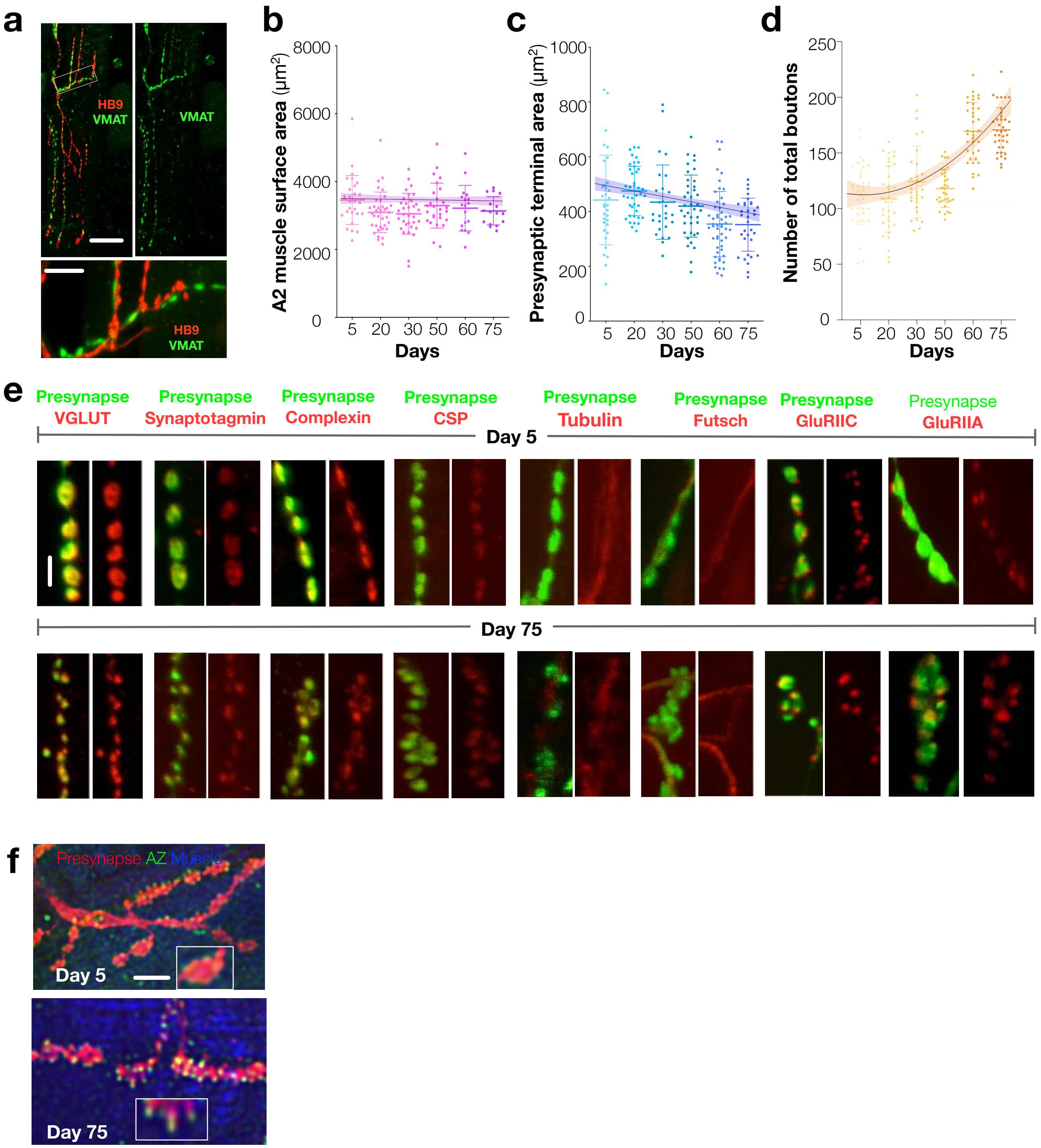

Supplementary Fig. 2. Measurement of active zone parameters, glutamate receptor puncta area and the ready releasable pool (RRP) of synaptic vesicles in young and old terminals.

a, b, Quantification of active zone (BRP) puncta size (a) and puncta intensity (b) in HB9 expressing terminals of young and old flies respectively.

c, Quantification of quantal content during ageing.

d, Quantification of dGluRIID puncta area in HB9-positive terminals of young, middle and old flies.

e, High frequency simulation of young and old terminals in elevated external Ca^{2+} revealing a significant reduction in the cumulative pool size and the estimated RRP size (in old flies versus young flies while the mEJC amplitudes remained unchanged between young and old flies. Note, estimated RRP size is much smaller than would be assumed from total AZ number. These data are explainable due to RRP measurements being made from one of six, electrically independent, muscle fibers. Thus, RRP estimates and for that matter, all physiological recordings, should be interpreted as measurements for single muscle fibers rather than total muscle field as analyzed in our morphological analysis.

Unpaired two-tailed *t*-test, ***p<0.001, n = 602 puncta per timepoint (a), *** p<0.001, $n \geq 4847$ puncta per timepoint (b), p = 0.08, $n \geq 6$ presynaptic terminals per timepoint (e, mEJC amplitude), ***p<0.001, $n \geq 11$ data points per timepoint (e, cumulative pool size), **p = 0.003, $n \geq 11$ data points per timepoint (e, estimated RRP size). One-way ANOVA, *p = 0.03, R^2 = 0.23, $n \geq 7$ presynaptic terminals per timepoint (c), ns=not significant, ***p<0.001, $n \geq 406$ puncta per timepoint (d), Tukey's multiple comparison tests were used to compare the mean of each time point with the mean of every other time point. Statistical analysis details with a precise value of '*n*' are reported in Supplementary Table 2 and in Source Data file. Data are represented as mean ± SD (c) and ± SEM (a, b, d and e). n = biologically independent samples.

Supplementary Fig. 2. Measurement of active zone parameters, glutamate receptor puncta area and the ready releasable pool (RRP) of synaptic vesicles in young and old terminals.

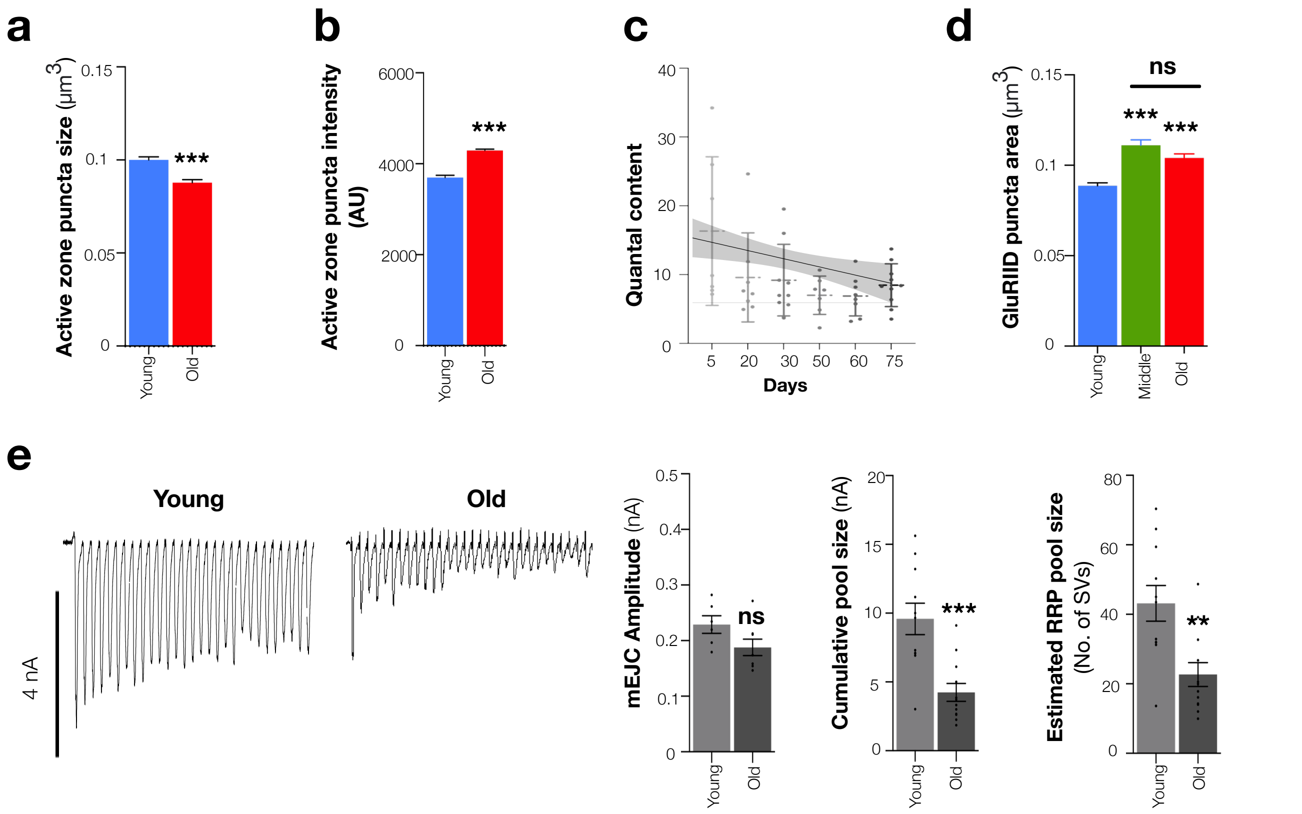

Supplementary Fig. 3*.* Synapse ageing parameters at alternate culturing temperatures.

a, Lifespan of control (UAS>GFP/+; HB9>Gal4/+) adult male flies at 18°C (blue; n=181), 25°C (green; n=292) and 29°C (red; n=336) culturing temperatures.

b, Duration of and percentage survival at the end of three stages (young, middle, old) when total lifespan is divided into three equal parts at 18°C, 25°C and 29°C culture temperatures.

c-h, Representative images of progressive degeneration of HB9 abdominal synaptic terminals (red, GFP) innervating A2 mvim muscles (blue, phalloidin) from 3 days after eclosion to 40 days cultured at 29°C. Magnification is identical for all images.

i-n, Representative images of progressive fragmentation of synaptic boutons (red, GFP) through reduction in size and number of active zones (green, Brp) per bouton during ageing cultured at 29°C. Magnification is identical for all images.

o, Bouton diameters decline progressively during ageing at 29°C**.**

p, Quantification of the number of active zones during ageing at 29°C.

q, Quantification of boutons with only a single active zone during ageing at 29°C.

r, Quantification of presynaptic terminal area during ageing at 29°C.

s-u, Quantification of EJC amplitude, mEJC amplitude and mEJC frequency of day 3 and day 35 old terminals at 29°C.

v, Quantification of boutons with only a single active zone during ageing at 18°C.

One-way ANOVA, ***p<0.001, $R^2 = 0.23$, $n = 150$ boutons per timepoint (o), $p = 0.07$, $R^2 = 0.16$, $n \geq 16$ presynaptic terminals per timepoint (p), ***p<0.001, $R^2 = 0.66$, $n \geq 17$ presynaptic terminals per timepoint (q), *$p = 0.02$, $R^2 = 0.10$, $n \geq 11$ presynaptic terminals per timepoint (r), ***p<0.001, $R^2 = 0.57$, $n \geq 9$ presynaptic terminals per timepoint (v), followed by Tukey's multiple comparison tests were used to compare the mean of each time point with the mean of every other time point. Statistical analysis details with a precise value of '*n*' are reported in Supplementary Table 3 and in Source Data file. Unpaired two-tailed *t*-test, **$p = 0.006$, $n \geq 13$ presynaptic terminals per timepoint (s, EJC amplitude), $p = 0.13$, ns = not significant. $n \geq 11$ presynaptic terminals per timepoint (t, mEJC amplitude), *$p = 0.04$, $n \geq 11$ presynaptic terminals per timepoint (u, mEJC frequency). Data are represented as mean ± SD (o, p, q, r and v) and ± SEM (s-u). n = biologically independent samples**.**

Supplementary Fig. 3. Synapse ageing parameters at alternate culturing temperatures.

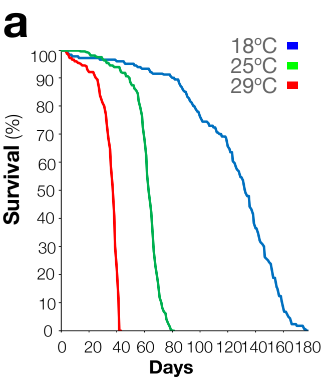

b

	18°C		25°C		29°C	
Ageing Period	Period length (days)	Survival at end of period (%)	Period length (days)	Survival at end of period (%)	Period length (days)	Survival at end of period (%)
Young	0-60	93.37	0-25	98.21	0-15	95.2
Middle	61-120	68.5	25-50	90.2	15-30	82.2
Old	120-180	0	50-75	0	30-45	0

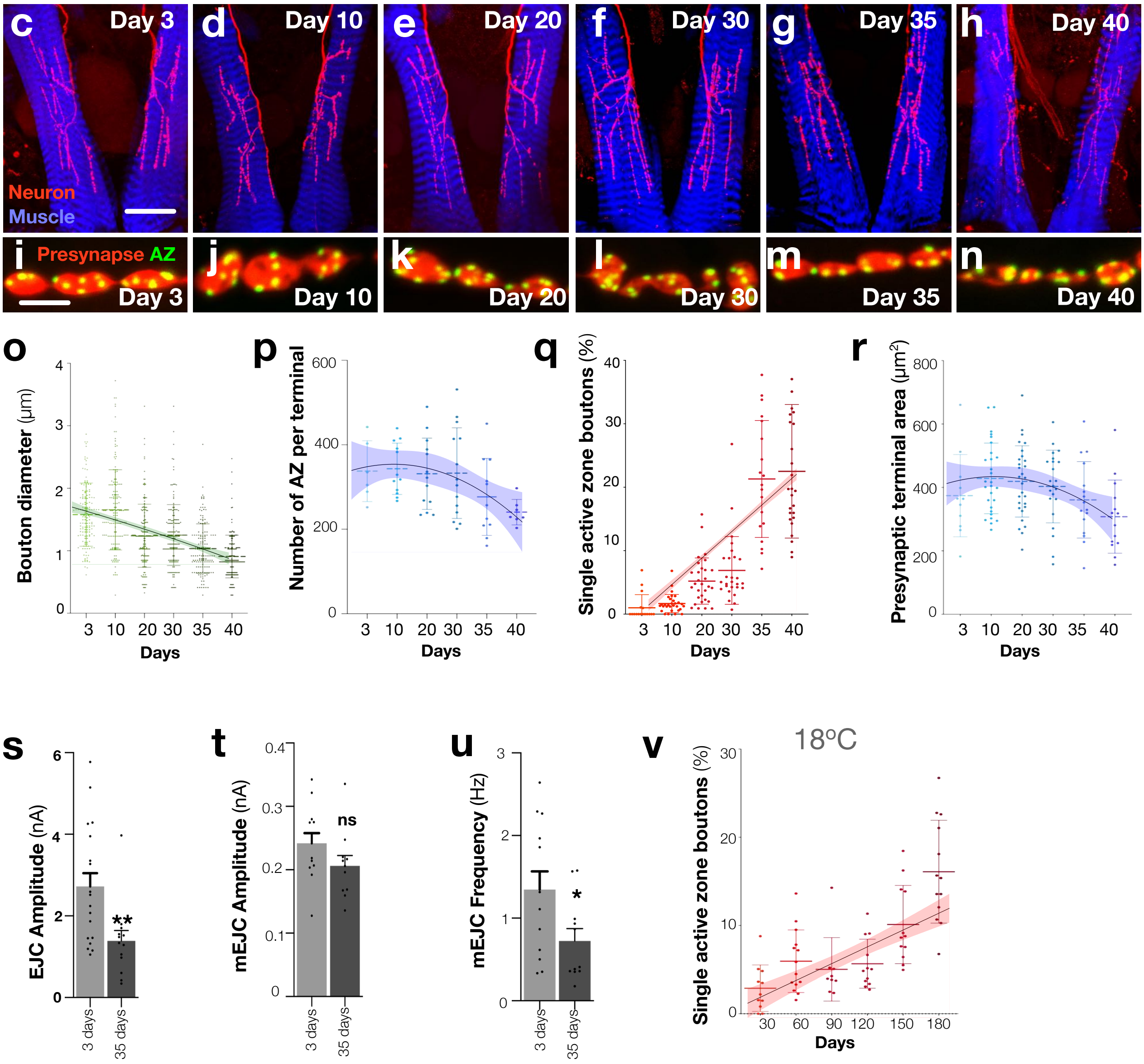

Supplementary Fig. 4. Validation and additional quantification of genetic reagents designed to deplete all vesicular or specifically evoked neurotransmission.

a, Schematic representation of genome engineered conditional excisable vglut. B3RT recognition sites were introduced to flank vglut coding exons in addition to introducing a LexA reporter element. In the absence of B3 recombinase, VGLUT is expressed and LexA is silent. Upon expression of B3, vglut coding exons are excised and LexA is expressed, positively marking the recombination event.

b, Representative VGLUT staining in HB9 and non-HB9 terminals at day 20. In control [UAS>GFP/+;HB9>Gal4,Tub>Gal80^{ts}], VGLUT staining (green) is observed at HB9 (red, GFP) and non-HB9 terminals. In *vglut-/-* mutants [Df(2R)371/B3RT_vglut_B3RT;HB9>Gal4,UAS>GFP,Tub>Gal80^{ts}/UAS>B3] VGLUT staining (green) is absent at HB9 expressing terminals (red, GFP) but still present at non-HB9 terminals.

c, Quantification of the number of active zones of control and *vglut-/-* mutants until day 35.

d, Quantification of the area of presynaptic terminals of control and *vglut-/-* mutants until day 20.

e, f, Quantification of boutons with only a single active zone (fragmentation) and number of active zones of non-HB9 presynaptic terminals of control and *vglut-/-* mutants until day 20.

g, Quantification of the number of active zones of control [UAS>luciferaseRNAi/HB9>Gal4, UAS>GFP,Tub>Gal80^{ts}] and HB9 neuron selective adult conditional Para knockdown mutants (ParaKD) [UAS>GFP/+;HB9>Gal4,Tub>Gal80^{ts},UAS>ParaRNAi] until day 35.

h, Quantification of the area of presynaptic terminals of control and ParaKD flies until day 20.

i, Representative EJC and mEJC traces, quantification of EJC amplitude, mEJC amplitude and mEJC frequency of control [UAS>luciferaseRNAi/HB9>Gal4,UAS>GFP,Tub>Gal80^{ts}] and ParaKD [UAS>GFP/+; HB9>Gal4,Tub>Gal80^{ts}/UAS>ParaRNAi] mutant flies at 35 days after eclosion.

j, Representative quantification of mEJC amplitude, mEJC frequency of Control [UAS>GFP/+; HB9>Gal4,Tub>Gal80^{ts}] and $Kir_{2.1E}$ expressing [UAS>$Kir_{2.1E}$;HB9>Gal4,UAS>GFP,Tub>Gal80^{ts}] terminals at 20 days.

k, Representative images of presynaptic HB9 neuron boutons (red, GFP) and active zones (Brp, green), quantification of bouton diameters and number of active zones and percentage of boutons with only a single active zone (bouton fragmentation) of control and $Kir_{2.1E}$ flies at 20 days after eclosion.

Two-way ANOVA, followed by Sidak's multiple comparison tests were used to compare the mean of control and experimental genotypes in that time point, ns=not significant, ***$p \leq 0.001$, $n \geq 9$ presynaptic terminals per timepoint (c), ns=not significant, *$p = 0.01$, $n \geq 17$ presynaptic terminals per timepoint (d), ns = not significant, $n \geq 11$ presynaptic terminals per timepoint (e and f), ns = not significant, $n \geq 7$ presynaptic terminals per timepoint (g), ns = not significant, $n \geq 12$ presynaptic terminals per timepoint (h). Unpaired two-tailed *t*-test, **$p = 0.004$, $n \geq 5$ presynaptic terminals per timepoint (i, EJC amplitude), $p = 0.79$, $n \geq 5$ presynaptic terminals per timepoint (i, mEJC amplitude), $p = 0.41$, $n \geq 5$ presynaptic terminals per timepoint (i, mEJC frequency), $p = 0.18$, $n \geq 7$ presynaptic terminals per timepoint (j, mEJC amplitude), $p = 0.83$, $n \geq 7$ presynaptic terminals per timepoint (j, mEJC frequency), $p = 0.09$, $n = 151$ boutons per timepoint (k, bouton diameters), $p = 0.44$, $n \geq 9$ presynaptic terminals per timepoint (k, number of active zones), $p = 0.80$, $n \geq 9$ presynaptic terminals per timepoint (k, single active zone boutons). All statistical analysis details with a precise value of '*n*' are reported in Supplementary Table 4 and 5 and in Source Data file. Experiments were carried out at 29°C. Scale bars, 30μm (b), 2μm (k). Data are represented as mean ± SEM. *n* = biologically independent samples.

Supplementary Fig. 4. Validation and additional quantification of genetic reagents designed to deplete all vesicular or specifically evoked neurotransmission.

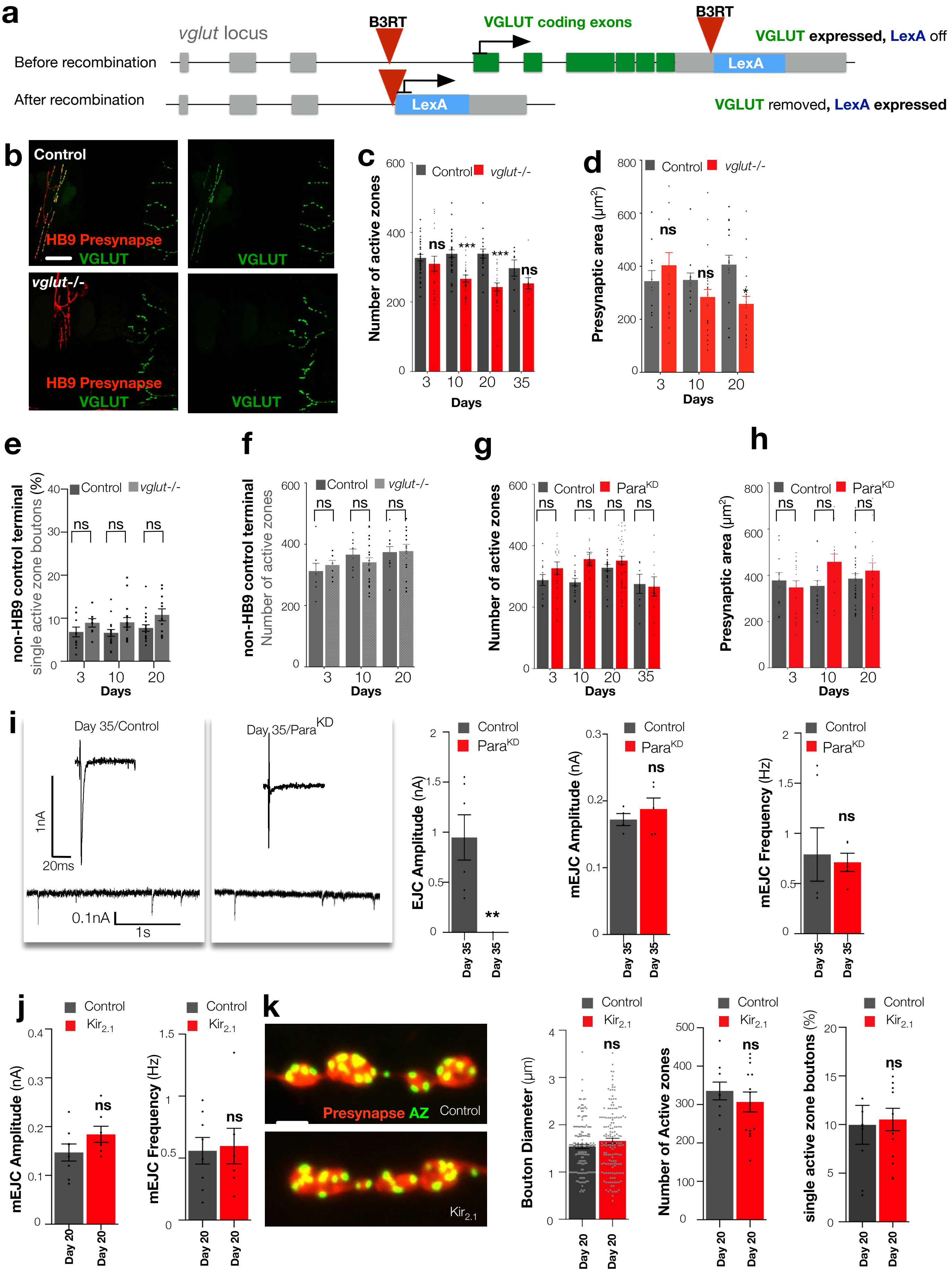

Supplementary Fig. 5. Validation and additional quantification of genetic reagents designed to specifically deplete miniature neurotransmission

a, Representative traces of EJC (above) and mEJC (below) of V100^{KD} [UAS>V100$^{3'UTR_RNAi}$; HB9>Gal4,UAS>GFP,TubGal80^{ts}] terminals at day 20. Quantification of EJC amplitude, mEJC amplitude and mEJC frequency of control [UAS>luciferase^{RNAi}/ HB9>Gal4,UAS>GFP,Tub>Gal80^{ts}] and V100^{KD}[UAS>V100$^{3'UTR_RNAi}$; HB9>Gal4,UAS>GFP,TubGal80^{ts}] terminals at day 20.

b, Representative anti-V100 immunostaining at day 20 in control [UAS>GFP/+; HB9>Gal4,Tub>Gal80^{ts}],V100^{KD}[UAS>V100$^{3'UTR_RNAi}$;HB9>Gal4,UAS>GFP,TubGal80^{ts}], rescued V100^{wt} [UAS>V100$^{3'UTR_RNAi}$/UAS>V100^{WT};HB9>Gal4,UAS>GFP,TubGal80^{ts}] and rescued V100^{WFI} [UAS>V100$^{3'UTR_RNAi}$/UAS>V100^{WFI}; HB9>Gal4,UAS>GFP,TubGal80^{ts}] terminals at day 20.

c, Quantification of the number of active zones of V100^{WT} [UAS>V100$^{3'UTR_RNAi}$/UAS>V100^{WT}; HB9>Gal4,UAS>GFP,TubGal80^{ts}] and V100^{WFI} [UAS>V100$^{3'UTR_RNAi}$/UAS>V100^{WFI}; HB9>Gal4,UAS>GFP,TubGal80^{ts}] mutant terminals until day 35.

d, Quantification of the area of presynaptic terminals of V100^{WT} and V100^{WFI} mutants until day 20.

e, f, Quantification of bouton fragmentation and number of active zones of non-HB9 presynaptic terminals of V100^{WT} and V100^{WFI} mutants until day 20.

g, Representative EJC and mEJC traces, quantification of EJC amplitude, mEJC amplitude and mEJC frequency of Control [UAS>luciferase^{RNAi} / HB9>Gal4, UAS>GFP,Tub>Gal80^{ts}] and V100^{WFI} + Para^{KD} [UAS>V100$^{3'UTR_RNAi}$/UAS>V100^{WFI}; HB9>Gal4,Tub>Gal80^{ts}/UAS>Para^{RNAi}] mutant flies at 20 days.

h, Representative images of boutons (red, GFP) and active zones (Brp, green), quantification of bouton diameters and number of active zones and percentage of boutons with only a single active zone (bouton fragmentation) Control [UAS>luciferase^{RNAi}/ HB9>Gal4,UAS>GFP,Tub>Gal80^{ts}], V100^{WFI} + Para^{KD} [UAS>V100$^{3'UTR_RNAi}$/UAS>V100^{WFI}; HB9>Gal4,UASGFP>Tub>Gal80^{ts}/UAS>Para^{RNAi}] and V100^{WFI} + Luciferase^{RNAi} [UAS>V100$^{3'UTR_RNAi}$/UAS>V100^{WFI}; HB9>Gal4,UASGFP>Tub>Gal80^{ts}/ UAS>luciferase^{RNAi}] at 20 days after eclosion.

Unpaired two-tailed *t*-test, ***$p \leq 0.001$, $n \geq 7$ presynaptic terminals per timepoint (a, EJC amplitude), $p = 0.37$, $n \geq 9$ presynaptic terminals per timepoint (a, mEJC amplitude), *$p = 0.04$, n ≥ 9 presynaptic terminals per timepoint (a, mEJC frequency), **$p = 0.007$, $n \geq 7$ presynaptic terminals per timepoint (g, EJC amplitude), $p = 0.36$, $n \geq 5$ presynaptic terminals per timepoint (g, mEJC amplitude), ***$p \leq 0.001$, $n \geq 5$ presynaptic terminals per timepoint (g, mEJC frequency). Two-way ANOVA, followed by Sidak's multiple comparison tests were used to compare the mean of control and experimental genotypes in that time point, ns=not significant, *$p \leq 0.03$, $n \geq 8$ presynaptic terminals per timepoint (c), ns=not significant, **$p \leq 0.008$, $n \geq 11$ presynaptic terminals per timepoint (d), ns = not significant, $n \geq 7$ presynaptic terminals per timepoint (e and f). One-way ANOVA plus Tukey's multiple comparisons test, ***$p \leq 0.001$, $n = 150$ boutons per timepoint (h, bouton diameters), ns=not significant, $n \geq 12$ presynaptic terminals per timepoint (h, number of active zones), **$p = 0.01$ (Control *vs* V100^{WFI} + Para^{KD}) **$p = 0.3$ (Control *vs* V100^{WFI} + Luciferase^{RNAi}), $n \geq 11$ presynaptic terminals per timepoint (h, Single active zone boutons). All statistical analysis details with a precise value of '*n*' are reported in Supplementary Table 4 and 5 and in Source Data file. Experiments were carried out at 29°C. Scale bars, 5μm (b), 2μm (h). Data are represented as mean ± SEM. n = biologically independent samples.

Supplementary Fig. 5. Validation and additional quantification of genetic reagents designed to specifically deplete miniature neurotransmission

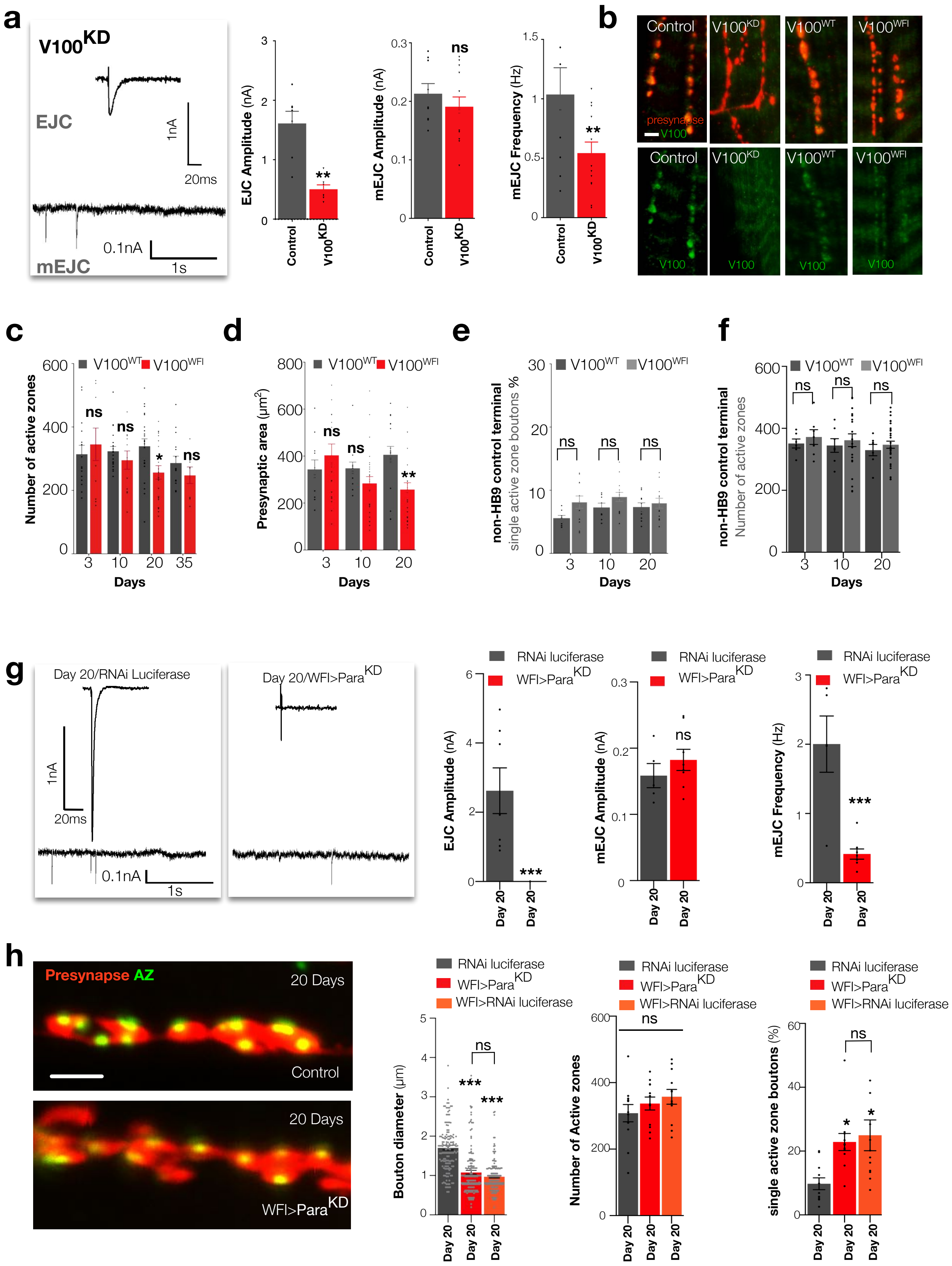

Supplementary Fig. 6. Presynaptic Homeostatic Potentiation at adult NMJ terminals

a, Representative EJC and mEJC traces, quantification of EJC amplitude, mEJC amplitude and quantal content of wild type and GluRIIA$^{-/-}$ (dGlurIIA^{JA1}/ dGlurIIA^{JA1}; HB9>Gal4,UAS>GFP/+) terminals at 20 days.

b, Representative images of boutons (red, GFP) and active zones (Brp, green), quantification of bouton diameters and percentage of boutons with only a single active zone (bouton fragmentation) and total number of active zones of wild type and GluRIIA$^{-/-}$ mutants [dGlurIIA^{JA1}/ dGlurIIA^{JA1}; HB9>Gal4,UAS>GFP] at 20 days. Unpaired two-tailed *t*-test, $p = 0.54$, $n \geq 11$ presynaptic terminals per timepoint (a, EJC amplitude), *$p = 0.02$, $n \geq 10$ presynaptic terminals per timepoint (a, mEJC amplitude), *$p = 0.04$, $n \geq 11$ presynaptic terminals per timepoint (a, quantal content), $p = 0.30$, $n = 151$ boutons per timepoint (b, bouton diameters), $p = 0.80$, $n \geq 10$ presynaptic terminals per timepoint (b, number of active zones), $p = 0.64$, $n \geq 10$ presynaptic terminals per timepoint (b, single active zone boutons). All statistical analysis details with a precise value of '*n*' are reported in Supplementary Table 4 and in Source Data file. Experiments were carried out at 29°C. Scale bars, 2 μm(b). Data are represented as mean ± SEM. *n* = biologically independent samples.

Supplementary Fig. 6. Presynaptic Homeostatic Potentiation at adult NMJ terminals

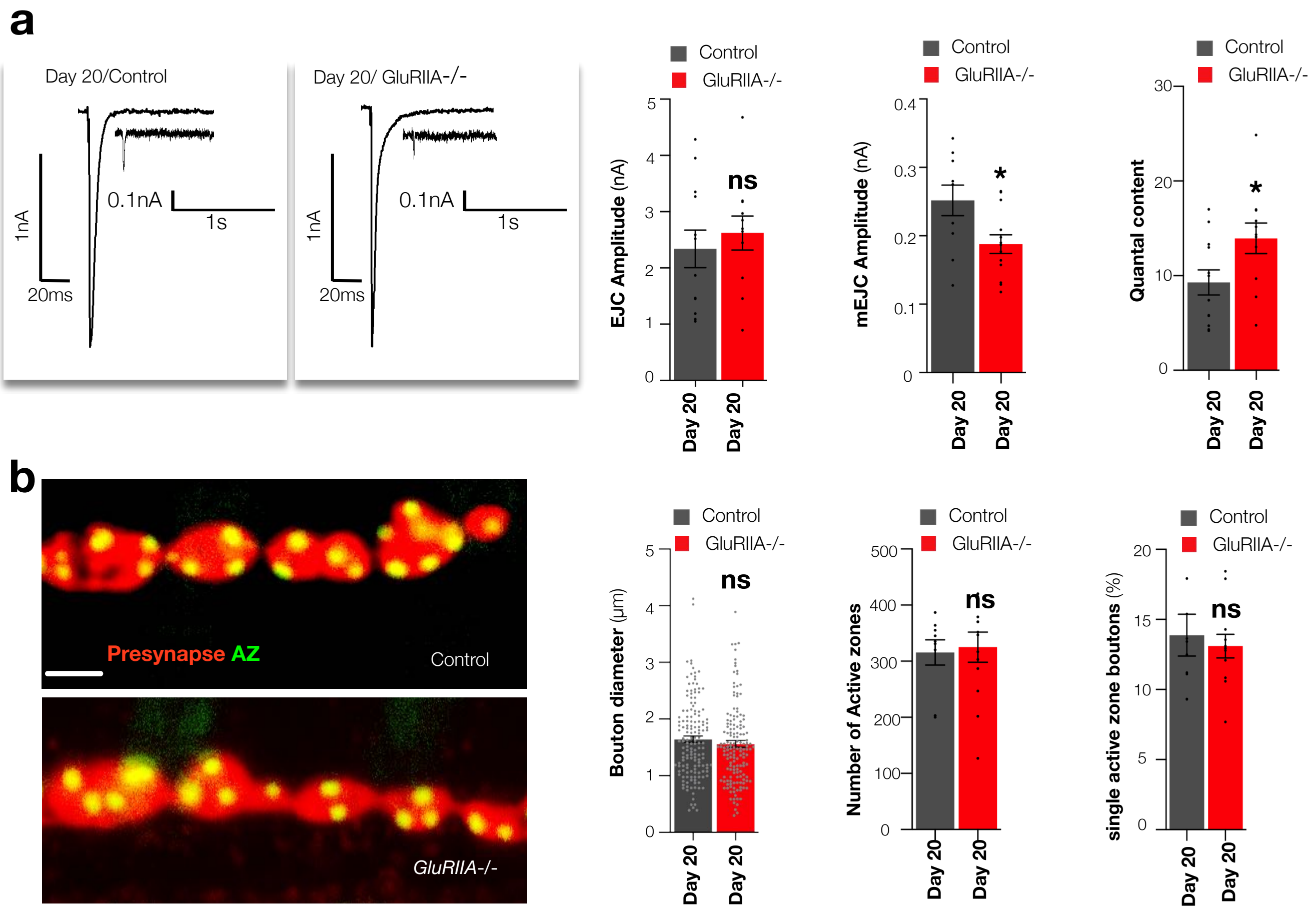

Supplementary Fig. 7. Validation and additional quantification of genetic reagents designed to specifically increase miniature neurotransmission.

a, Representative Cpx staining at HB9 and non-HB9 terminals at day 20. In control [UAS>GFP/+; HB9>Gal4,Tub>Gal80^{ts}], Cpx staining (green) is observed at HB9 (red) and non-HB9 terminals. In Cpx^{KD} [Cpx^{RNAi} /+; Hb9-Gal4,UAS>GFP,TubGal80^{ts}] animals, Cpx staining is present at non-HB9 terminals, and it is absent at HB9 terminals (red).

b, Representative traces of EJC (above) and mEJC (below) of control, Cpx^{KD} and Cpx^{KD};Para^{KD} terminals at day 20. Quantification of EJC amplitude, mEJC amplitude and mEJC frequency of control [UAS>luciferase^{RNAi}/HB9>Gal4,UAS>GFP,Tub>Gal80^{ts}], Cpx^{KD} [UAS-Cpx^{RNAi} /+; HB9-Gal4,UAS>GFP,TubGal80^{ts}] and Cpx^{KD};Para^{KD} [UAS-Cpx^{RNAi} /+; HB9-Gal4,UAS>GFP,TubGal80^{ts}/Para^{RNAi}] terminals at day 20.

c, Examples of transgenic expressed myc-tagged mCpx^{HB} (green) in HB9-positive presynaptic terminals (red, GFP) of mCpx^{HB} [UAS>Cpx^{RNAi}/UAS>GFP; HB9>Gal4,Tub>Gal80^{ts}/*cpx*^{SH1},UAS>mCpx^{HB}] mutants.

d, e, Quantification of number of active zone and presynaptic terminal area of mCpx^{WT} [UAS> Cpx^{RNAi} /UAS>GFP; HB9>Gal4,TubGal80^{ts} / *cpx*^{SH1},UAS>mCpx^{WT}] and mCpx^{HB} [UAS>Cpx^{RNAi} /UAS>GFP; HB9>Gal4,Tub>Gal80^{ts} / *cpx*^{SH1}, UAS>mCpx^{HB}] mutants during ageing.

f, Quantification of number of active zones and percentage of boutons with only a single active zone (bouton fragmentation) of Control, Cpx^{KD} and Cpx^{KD};Para^{KD} mutant flies at 35 days after eclosion.

One-way ANOVA plus Tukey's multiple comparisons test, $^*p = 0.02$ (Control *vs* Cpx^{KD}), $^{***}p \leq 0.001$ (Control *vs* Cpx^{KD};Para^{KD}), $^*p = 0.02$ (Cpx^{KD} *vs* Cpx^{KD};Para^{KD}), $n \geq 6$ presynaptic terminals per timepoint (b, EJC amplitude), ns = not significant, $n \geq 10$ presynaptic terminals per timepoint (b, mEJC amplitude), $^*p = 0.02$ (Control *vs* Cpx^{KD}), $^{***}p \leq 0.001$ (Control *vs* Cpx^{KD};Para^{KD}), $p = 0.27$ (Cpx^{KD} *vs* Cpx^{KD};Para^{KD}), $n \geq 10$ presynaptic terminals per timepoint (b, mEJC frequency), $^{***}p \leq 0.001$ (Control *vs* Cpx^{KD}), $^{**}p = 0.004$ (Control *vs* Cpx^{KD};Para^{KD}), $p = 0.15$ (Cpx^{KD} *vs* Cpx^{KD};Para^{KD}), $n \geq 11$ presynaptic terminals per timepoint (f, number of active zones), $^*p = 0.05$ (Control *vs* Cpx^{KD}), $^*p = 0.04$ (Control *vs* Cpx^{KD};Para^{KD}), $p = 0.88$ (Cpx^{KD} *vs* Cpx^{KD};Para^{KD}), $n \geq 11$ presynaptic terminals per timepoint (f, single active zone boutons). Two-way ANOVA, followed by Sidak's multiple comparison tests, $^*p \leq 0.03$, $^{**}p \leq 0.002$, ns= not significant, $n \geq 6$ presynaptic terminals per timepoint (d), $^{**}p \leq 0.002$, ns= not significant, $n \geq 7$ presynaptic terminals per timepoint (e). All statistical analysis details with a precise value of '*n*' are reported in Supplementary Table 4 and 6 and in Source Data file. Experiments were carried out at 29°C. Scale bars, 30μm (a and c). Data are represented as mean ± SEM. n = biologically independent samples.

Supplementary Fig. 7. Validation and additional quantification of genetic reagents designed to specifically increase miniature neurotransmission.

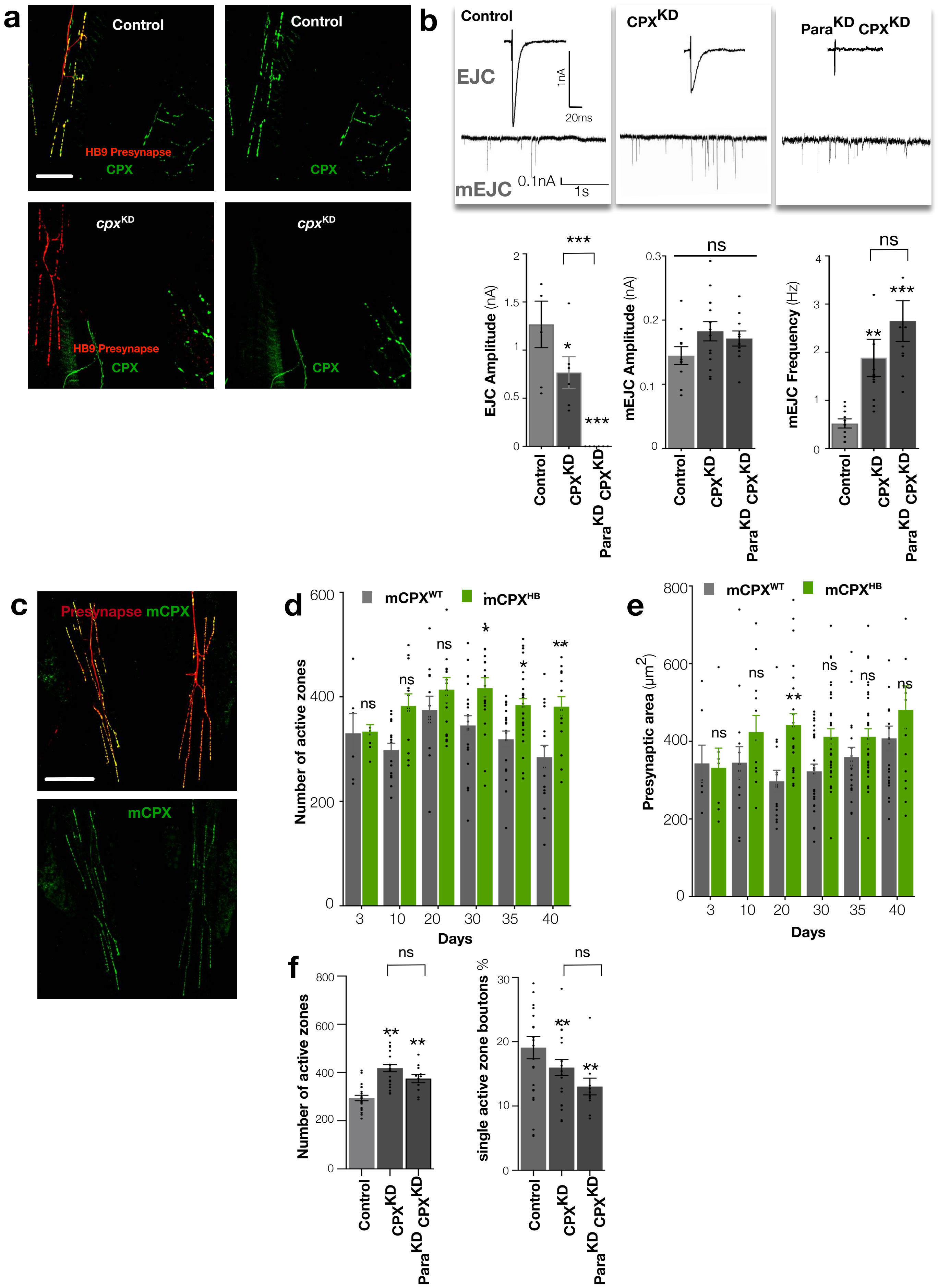

Supplementary Table 1: Genotypes for Fig. 1 and Supplementary Fig. 1-3

Figures	Genotypes
Fig. 1 d-r,u,v<br>Fig. 2a-f<br>Supplementary Fig. 1a-e<br>Supplementary Fig. 2a-e<br>Supplementary Fig. 3a-v	UAS>GFP/+; HB9>Gal4/+
Fig. 1s	BRP-GFP/+; HB9>Gal4,UAS>Cherry/+
Fig. 1t	Repo>QF2/+; HB9>Gal4,UAS>GFP/QUAS>Cherry
Supplementary Fig. 1f	Ok6>Gal4/ BRP-GFP; UAS>Cherry/+

Supplementary Table 2: Morphological and electrophysiological analyses of HB9 motor neuron terminals at A2 mvim during ageing at 25°C

Synapse Parameters	Day 5	Day 20	Day 30	Day 50	Day 60	Day 75
Total boutons	113.11±4.72 (34)	108.61±5.08 (34)	124.06±5.97 (29)	117.90.06±2.91 (33)	169.40±4.23 (37)<br>*(p< 0.001 vs day 5)****<br>*(p< 0.001 vs day 25)****<br>*(p< 0.001 vs day 30)****<br>*(p< 0.001 vs day 50)****	170.67±3.35 (37)<br>*(p< 0.001 vs day 5)****<br>*(p< 0.001 vs day 25)****<br>*(p< 0.001 vs day 30)****<br>*(p< 0.001 vs day 50)****
Single active zone boutons /total boutons	0.09±0.008 (34)	0.06±0.01 (34)	0.09±0.01 (29)	0.14±0.01 (33)<br>*(p=0.006 vs day 20)***	0.18±0.01 (37)<br>*(p< 0.001 vs day 5)****<br>*(p< 0.001 vs day 20)****<br>*(p< 0.001 vs day 30)****	0.31±0.02 (36)<br>*(p< 0.001 vs day 5)****<br>*(p< 0.001 vs day 20)****<br>*(p< 0.001 vs day 30)****<br>*(p< 0.001 vs day 50)****<br>*(p< 0.001 vs day 60)****
Average bouton diameter (um)	2.162±0.068 (150)	1.952±0.075 (150)	1.80±0.069 (150)<br>*(p=0.002 vs day 5)***	1.83±0.078 (150)<br>*(p=0.008 vs day 5)***	1.04±0.061 (150)<br>*(p< 0.001 vs day 5)****<br>*(p< 0.001 vs day 20)****<br>*(p< 0.001 vs day 30)****<br>*(p< 0.001 vs day 50)****	0.782±0.037 (150)<br>*(p< 0.001 vs day 5)****<br>*(p< 0.001 vs day 20)****<br>*(p< 0.001 vs day 30)****<br>*(p< 0.001 vs day 50)****
Presynaptic area (um^2)	442.32±28.5 (33)	475.21±14.19 (40)	457.08±34.53 (27)	419.97±18.18 (39)	354.51±16.31 (53)<br>*(p=0.014 vs day 5)**<br>*(p< 0.001 vs day 20)****	352.31±16.8 (33)<br>*(p=0.031 vs day 5)**<br>*(p=0.0003 vs day 20)****
Number of active zones	326.1±15.3 (21)	364±8.54 (27)	384±11.71 (34)	361.2±26.23 (25)	322.3±17.04 (23)	291.1±15.89 (38)<br>*(p=0.012 vs day 20)**<br>*(P<0.001 vs day 30)****<br>*(p=0.02 vs day 50)**
A2 Muscle area (um^2)	3459.56±136.07 (28)	3092.38±99.71 (36)	3052.57±104.54 (33)	3292.26±127.32 (27)	3218.33±157.85 (18)	3136.42±103.89 (16)
Evoked (EJC) Amplitude) (nA)	3.37±0.84 (7)	1.8±0.43 (8)	1.83±0.34 (9)	1.51±0.22 (7)<br>*(P=0.04 vs day 5)**	1.37±0.2 (8)<br>*(p=0.02 vs day 5)**	1±0.11 (10)<br>*(p=0.001 vs day 5)***
Miniature (mEJC) Frequency (Hz)	1.01±0.11 (20)	0.85±0.12 (18)	0.75±0.12 (15)	0.90±0.12 (17)	0.49±0.08 (17)<br>*(p=0.01 vs day 5)**	0.498±0.08 (13)<br>*(p=0.03 vs day 5)**
Miniature (mEJC) Amplitude (nA)	0.20±0.01 (20)	0.18±0.014 (18)	0.199±0.014 (15)	0.193±0.017 (17)	0.20±0.028 (17)	0.118±0.016 (13)<br>*(p=0.03 vs day 5)**
Quantal content	16.32±3.66 (8)	9.58±2.08 (9)	9.19±1.59 (10)	7±0.94 (8)<br>*(p=0.04 vs day 5)**	6.89±0.92 (9)<br>*(p=0.03 vs day 5)**	8.46±0.9 (11)
Input resistance (MΩ)	24.67±1.41 (20)	27.55±1.58 (18)	25.46±1.46 (15)	23.22±1.75 (18)	19.36±1.41 (17)<br>*(p=0.007 vs day 20)***	17.65±2.43 (13)<br>*(p=0.001 vs day 20)***<br>*(p=0.03 vs day 30)**
Climbing ability %	95.35 ± 1.28 (10)	79.12 ± 4.35 (9)	50.34 ± 5.99 (11)<br>*(p< 0.001 vs day 5)****<br>*(p< 0.001 vs day 20)****	29.97 ± 4.18 (10)<br>*(p< 0.001 vs day 5)****<br>*(p< 0.001 vs day 20)****<br>*(p=0.01 vs day 30)**	10.37 ± 3.35 (6)<br>*(p< 0.001 vs day 5)****<br>*(p< 0.001 vs day 20)****<br>*(p< 0.001 vs day 30)****	0 ± 0 (5)<br>*(p< 0.001 vs day 5)****<br>*(p< 0.001 vs day 20)****<br>*(p< 0.001 vs day 30)****<br>*(p=0.002 vs vs day 50)***

Statistical significance was tested by Ordinary One-way ANOVA followed by Tukey's multiple comparisons test, **p*< 0.033 ** *p*< 0.002, *** *p*< 0.001

Supplementary Table 3: Morphological and electrophysiological analyses of HB9 motor neuron terminals at A2 mvim during ageing at alternate culturing temperatures

Synapse Parameters	**Day 3**	**Day 10**	**Day 20**	**Day 30**	**Day 35**	**Day 40**
Average bouton diameter (um)	1.57±0.04 (150)	1.65±0.05 (150)	1.23±0.04(150) *(p< 0.001 vs day 3)**** *(p< 0.001 vs day 10)****	1.25±0.03(150) *(p< 0.001 vs day 3)**** *(p< 0.001 vs day 10)****	1.03±0.03 (150) *(p< 0.001 vs day 3)**** *(p< 0.001 vs day 10)**** *(p= 0.004 vs day 20)**** *(p= 0.002 vs day 30)****	0.9±0.02 (150) *(p< 0.001 vs day 3)**** *(p< 0.001 vs day 10)**** *(p< 0.001 vs day 20)**** *(p< 0.001 vs day 30)****
Number of active zones	337.1±28.03 (6)	343.2±17.17 (12)	331.2±24.93 (13)	332.2±28.1 (14)	276.7±26.8 (11)	240.5±10.9 (7)
Presynaptic area (um^2)	374.1±40 (11)	428.48±20.82 (28)	419.16.1±20.66 (29)	403.1±24.12 (22)	360.97±29.66 (16)	307.85±30.28 (14) *(p= 0.02 vs day 10)** *(p= 0.04 vs day 20)**
Single active zone boutons /total boutons	0.01±0.004 (17)	0.01±0.001 (30)	0.05±0.004 (30)	0.06±0.005 (27) *(p= 0.03 vs day 3)** *(p= 0.02 vs day 10)**	0.21±0.02 (19) *(p< 0.001 vs day 3)**** *(p< 0.001 vs day 10)**** *(p< 0.001 vs day 20)**** *(p< 0.001 vs day 30)****	0.22±0.01 (26) *(p< 0.001 vs day 3)**** *(p< 0.001 vs day 10)**** *(p< 0.001 vs day 20)**** *(p< 0.001 vs day 30)****
Evoked (EJC) Amplitude) (nA)	2.72±0.38 (19)	Not recorded	Not recorded	Not recorded	1.38±0.20 (13) *(p= 0.006 vs day 3)***	Not recorded
Miniature (mEJC) Amplitude (nA)	0.24±0.01 (13)	Not recorded	Not recorded	Not recorded	0.20±0.01 (11)	Not recorded
Miniature (mEJC) Frequency (Hz)	1.34±0.21 (13)	Not recorded	Not recorded	Not recorded	0.72±0.14 (11) *(p= 0.02 vs day 3)**	Not recorded
Synapse Parameters at 18°C	**Day 30**	**Day 60**	**Day 90**	**Day 120**	**Day 150**	**Day 180**
Single active zone boutons /total boutons at 18°C	0.02±0.007 (11)	0.05±0.009 (14)	0.05±0.011 (9)	0.05±0.007 (12)	0.10±0.012 (12) *(p< 0.001 vs day 30)****	0.16±0.01 (13) *(p< 0.001 vs day 30)**** *(p< 0.001 vs day 60)**** *(p< 0.001 vs day 90)**** *(p< 0.001 vs day 120)**** *(p= 0.005 vs day 150)****

Statistical significance was tested by Ordinary One-way ANOVA followed by Tukey's multiple comparisons test, **p*< 0.033 ** *p*< 0.002, *** *p*< 0.001

Supplementary Table 4: Electrophysiological measurements of indicated genotypes in adult HB9 motor neuron terminals at 29ºC

Genotypes (Aged day 20)	*Evoked (EJC) Amplitude (nA)*	*Miniature (mEJC) Frequency (Hz)*	*Miniature (mEJC) Amplitude (nA)*
**(A) Background Control**<br>*UAS>GFP/+ ;HB9>Gal4,Tub>Gal80*^{ts}	1.44±0.21 (10)	0.87±0.16 (17)	0.16±0.01 (17)
**(B) Inhibition of EJC and mEJC (*vglut*$^{-/-}$)**<br>*Df(2R)371/B3RT_vglut_B3RT; HB9>Gal4,UAS>GFP,Tub>Gal80*^{ts}	0.091±0.03 (9)<br>(*p*< 0.001 vs A)***	0.12±0.02 (17)<br>(*p*< 0.001 vs A)***	0.18±0.01 (17)
**(C) Inhibition of EJC (Para^{KD})**<br>*UAS>GFP/+; HB9>Gal4,Tub>Gal80*^{ts}*/UAS>Para*^{RNAi}	0±0 (6)<br>(*p*=0.001 vs D)**	1.04±0.14 (10)	0.13±0.01 (10)
**(D) RNAi Control**<br>*UAS>luciferase*^{RNAi} */ HB9>Gal4, UAS>GFP,Tub>Gal80*^{ts}	1.8±0.36 (8)	0.86±0.21 (10)	0.16±0.009 (10)
**(E) Inhibition of mEJC (V100^{WFI})**<br>*UAS-V100*^{WFI}*/ UAS-V100RNAi; Hb9-Gal4, UAS>GFP,TubGal80*^{ts}	1.58±0.4 (9)	0.17±0.04 (8)<br>(*p*=0.004 vs F)**	0.19±0.01 (8)
**(F) Control mEJC inhibition (V100^{WT})**<br>*UAS>V100*$^{3'UTR_RNAi}$*/UAS>V100*^{WT}*; HB9>Gal4,UAS>GFP,TubGal80*^{ts}	2.09±0.3 (8)	0.98±0.2 (11)	0.15±0.01 (11)
**(G) CPX^{KD}**<br>*Cpx*^{RNAi} */+; Hb9-Gal4,UAS>GFP,TubGal80*^{ts}*/* ^{SH1}	0.76±0.16 (9)<br>(*p*=0.006 vs D)**	1.7±0.36 (14)<br>(*p*=0.04 vs D)**	0.18±0.01 (14)
**(H) Increase mEJC (mCpx^{HB})**<br>*UAS>Cpx*^{RNAi} */UAS>GFP; HB9>Gal4,Tub>Gal80*^{ts} */ cpx*^{SH1}*, UAS>mCpx*^{HB}	1.21±0.21 (6)	2.35±0.29 (16)<br>(*p*=0.003 vs I)**	0.15±0.01 (16)
**(I) Control mEJC increase (mCpx^{WT})**<br>*UAS> Cpx*^{RNAi}*/UAS>GFP; HB9>Gal4,TubGal80*^{ts} */ cpx*^{SH1}*,UAS>mCpx*^{WT}	1.2±0.24 (8)	1.13±0.16 (15)	0.15±0.01 (15)
**(J) Inhibition of EJC and mEJC (V100^{WFI}Para^{KD})**<br>UAS>V100$^{3'UTR_RNAi}$/UAS>V100^{WFI}; HB9>Gal4,Tub>Gal80^{ts}/UAS>Para^{RNAi}	0±0 (5)	0.41±0.07 (8)	0.18±0.01 (8)
**(K) Increase mEJC and decrease EJC (CPX^{KD}Para^{KD})**<br>UAS- Cpx^{RNAi} /+; Hb9-Gal4,UAS>GFP,TubGal80^{ts}/Para^{KD}	0±0 (6)	2.64±0.42 (10)<br>(*p*=0.003 vs I)**	0.17±0.01 (10)
**(L) Inhibition of EJC (Kir$_{2.1}$)**<br>UAS> Kir$_{2.1}$; HB9>Gal4,UAS>GFP,Tub>Gal80^{ts}	na	0.6±0.14 (7)	0.18±0.01 (7)
**(M) presynaptic homeostatic potentiation (GluRIIA$^{-/-}$)**	2.62±0.3 (11)	na	0.18±0.01 (14)
**(N) Control for presynaptic homeostatic potentiation (W^{118})**	2.34±0.3 (12)	na	0.25±0.02 (11)

Statistical significance was tested by Unpaired *t*-test. ***p*< 0.033 ** *p*< 0.002, *** *p*< 0.001

Supplementary Table 5: Morphological measurements of indicated genotypes at indicated age at 29°C

	Synapse parameters	**(A)** **Background Control** *UAS>GFP/+ ;HB9>Gal4,Tub>Gal80^{ts}*	**(B)** **Inhibition of EJC and mEJC** *Df(2R)371/B3RT_vglut_B3RT; HB9>Gal4,UAS>GFP,Tub>Gal80^{ts}*	**(C)** **Inhibition of EJC** Para^{KD} (sodium channel) *UAS>GFP/+; HB9>Gal4,Tub>Gal80^{ts}/UAS>Para^{RNAi}*	**(D)** **RNAi Control** *UAS>luciferase^{RNAi} / HB9>Gal4, UAS>GFP,Tub>Gal80^{ts}*	**(E)** **Inhibition of mEJC (V100^{WFI})** *UAS>V100$^{3'UTR_RNAi}$/UAS>V100^{WFI}; HB9>Gal4,UAS>GFP,TubGal80^{ts}*	**(F)** **Control inhibition of mEJC (V100^{WT})** *UAS>V100$^{3'UTR_RNAi}$/UAS>V100^{WT}; HB9>Gal4,UAS>GFP,TubGal80^{ts}*
Day 3	*Number of boutons with only a single active zone/total boutons*	0.06±0.006 (31)	0.072±0.008 (16)	0.05±0.01 (11)	0.06±0.008 (12)	0.07±0.006 (21)	0.05±0.01 (17)
	Average bouton diameter (um)	1.31±0.04 (150)	1.37±0.06 (150)	1.29±0.039 (150)	1.34±0.03 (150)	1.51±0.06 (150)	1.44±0.04 (150)
	Presynaptic area (um^2)	394.53±21.51 (41)	398.52±37.35 (17)	348.65±28.31 (17)	378.77±33.5 (12)	403.7±50.47(18)	358.23±39.03 (11)
	Number of active zones	327.41±9.39 (31)	310.36±21.58 (19)	327.05±20.26 (17)	288.3±17.1 (15)	345.6±51.25 (10)	314.46±27.94 (15)
Day 10	*Number of boutons with only a single active zone/total boutons*	0.08±0.007 (41)	0.28±0.02 (23) (*p*<0.001 vs A)***	0.06±0.005 (24)	0.09±0.013 (23)	0.2±0.012 (34) (*p*<0.001 vs F)***	0.1±0.01 (34)
	Average bouton diameter (um)	1.51±0.07 (150)	1.07±0.03 (150) (*p*<0.001 vs A)***	1.5±0.04 (150)	1.73±0.06 (150)	0.92±0.03 (150) (*p*<0.001 vs F)***	1.56±0.05 (150)
	Presynaptic area (um^2)	477.54±28.77 (30)	416.9±30.83 (26)	459.31±30.13 (15)	354.7±23.1 (18)	341.2±24.008 (26)	323.8±14.82 (19)
	Number of active zones	339.06±10.93 (30)	251.48±10.40 (28) (*p*=0.0009 vs A)***	356.94±21.98 (17)	285.4±10.3 (17)	295.66±28.83 (12)	348.6±25.8 (13)
Day 20	*Number of boutons with only a single active zone/total boutons*	0.08±0.008 (22)	0.41±0.03 (20) (*p*<0.001 vs A)***	0.08±0.008 (33)	0.07±0.006 (26)	0.25±0.01 (31) (*p*<0.001 vs F)***	0.16±0.01 (33)
	Average bouton diameter (um)	1.72±0.13 (150)	0.89±0.04 (150) (*p*<0.001 vs A)***	1.8±0.06 (150)	1.44±0.04 (150)	0.73±0.03 (150) (*p*<0.001 vs F)***	1.15±0.04 (150)
	Presynaptic area (um^2)	452.25±32.02 (35)	340.47±20.4 (26) (*p*=0.026 vs A)*	421.23±38.57 (25)	386.6±20.6 (33)	284.35±22.61 (24) (*p*=0.005 vs F)**	406.02±35.4 (18)
	Number of active zones	339.27±13.02 (18)	243.37±11.66 (24) (*p*=0.0001 vs A)***	352.02±13.74 (34)	327.3±9.1 (26)	256.76±21.31 (21) (*p*=0.017 vs F)*	339.7±22.8 (21)
Day 35	*Number of boutons with only a single active zone/total boutons*	0.17±0.01 (10)	0.37±0.03 (9) (*p*=0.0001 vs A)***	0.17±0.01 (10)	0.18±0.02 (7)	0.25±0.02 (14) (*p* = 0.02 vs F)*	0.2±0.02 (19)
	Average bouton diameter (um)	0.96±0.03 (150)	0.95±0.02 (150)	1.24±0.05 (150)	1.14±0.04 (150)	0.91±0.03 (150)	0.95±0.04 (150)
	Number of active zones	297.75±23.28 (8)	254±8.62 (10)	267.2±31.73 (10)	275.86±29.16 (8)	247.88±26.08 (8)	286.73±21.38 (14)

Statistical significance was tested by Two-way ANOVA followed by Sidak's multiple comparisons test, *$p< 0.033$ ** $p< 0.002$, *** $p< 0.001$

Supplementary Table 6: Morphological measurements of indicated genotypes at indicated age at 29°C

Days	*Synapse parameters*	**(A) RNAi Control** *UAS>luciferase^{RNAi} / HB9>Gal4, UAS>GFP,Tub>Gal80^{ts}*	**(B) Complexin^{KD}** *Cpx^{RNAi} /+; HB9-Gal4,UAS>GFP,TubGal80^{ts}/Cpx^{SH1}*	**(C) Increased mEJC (mCPX^{HB})** *UAS>Cpx^{RNAi} /UAS>GFP; HB9>Gal4,Tub>Gal80^{ts} / cpx^{SH1}, UAS>mCPX^{HB}*	**(D) Control Increased mEJC (mCPX^{WT})** *UAS> Cpx^{RNAi}/UAS>GFP; HB9>Gal4,TubGal80^{ts} / cpx^{SH1},UAS>mCPX^{WT}*
**Day 3**	*Number of boutons with only a single active zone/total boutons*	0.06±0.005 (15)	0.07±0.01 (7)	0.03±0.008 (8)	0.05±0.01 (7)
	Average bouton diameter (um)	1.58±0.06 (150)	1.52±0.04 (150)	1.73±0.05 (150)	1.55±0.04 (150)
	Presynaptic area (um^2)	314.71±19.09 (15)	393.84±29.05 (8)	306.17±29.05 (8)	343.12±37.93 (7)
	Number of active zones	280.05±16.46 (17)	339.14±23.32 (7)	333.62±50.82 (8)	330.33±47.01 (6)
**Day 10**	*Number of boutons with only a single active zone/total boutons*	0.067±0.008 (16)	0.08±0.008 (16)	0.06±0.011 (12)	0.08±0.008 (16)
	Average bouton diameter (um)	1.68±0.04 (150)	1.84±0.05 (150)	1.71±0.05 (150)	1.58±0.04 (150)
	Presynaptic area (um^2)	345.83±19.09 (25)	473.95±33.04 (24) (p=0.04 vs A)*	361.77±33.36 (12)	345.33±41.2 (14)
	Number of active zones	270.83±18.97 (12)	397.4±18.26 (20)	383.6±19.79 (14) (p=0.04 vs D)*	293±11.39 (20)
**Day 20**	*Number of boutons with only a single active zone/total boutons*	0.09±0.008 (24)	0.07±0.008 (24)	0.07±0.009 (22) (p=0.0009 vs D)***	0.16±0.02 (15)
	Average bouton diameter (um)	1.38±0.06 (150)	1.60±0.05 (150) (p=0.02 vs A)*	1.76±0.06 (150) (p=0.009 vs D)**	1.51±0.06 (150)
	Presynaptic area (um^2)	372.76±24.71 (18)	392.69±34.12 (24)	368.7±29.31 (22) (p=0.008 vs D)**	297.07±28.74 (15)
	Number of active zones	329.61±24.87 (18)	406.47±20.55 (17)	413.71±23.58 (21)	374.83±26.44 (12)
**Day 30**	*Number of boutons with only a single active zone/total boutons*	0.18±0.02 (28)	0.13±0.01 (26) (p=0.0048 vs A)**	0.1±0.009 (34) (p=0.0046 vs D)**	0.16±0.017 (29)
	Average bouton diameter (um)	1.38±0.06 (150)	1.67±0.05 (150) (p=0.001 vs A)***	1.63±0.06 (150) (p =0.0013 vs D)**	1.34±0.05 (150)
	Presynaptic area (um^2)	340.5±20.8 (24)	374.64±28.04 (22)	478.3±28.07 (32) (p =0.03 vs D)*	322.7±18.5 (28)
	Number of active zones	288.25±11.2 (20)	355.92±13.8 (27) (p =0.015 vs A)*	417.05±19.6 (20) (p=0.03 vs D)*	345.5±18.6 (28)
**Day 35**	*Number of boutons with only a single active zone/total boutons*	0.19±0.01 (26)	0.15±0.01 (21)	0.12±0.013 (30) (p<0.001 vs D)***	0.21±0.01(22)
	Average bouton diameter (um)	1.21±0.05 (150)	1.72±0.06 (150) (p<0.001 vs A)***	1.75±0.06 (150) (p<0.001 vs D)***	0.95±0.04 (150)
	Presynaptic area (um^2)	331.3±26.9 (27)	459.4±38.9 (23) (p=0.01 vs A)*	411.5±20.8 (32)	359.5±25.05 (21)
	Number of active zones	294.28±11.4 (21)	418.3±14.5 (27) (p<0.001 vs A)***	384.03±12.71 (28) (p=0.04 vs D)*	319.2±15.5 (19)
**Day 40**	*Number of boutons with only a single active zone/total boutons*	0.26±0.013 (46)	0.14±0.01(34) (p=0.0002 vs A)***	0.13±0.012 (28) (p=0.0007 vs D)***	0.21±0.01 (19)
	Average bouton diameter (um)	1.19±0.06 (150)	1.57±0.04 (150) (p<0.001 vs A)***	1.59±0.06 (150) (p<0.001 vs D)***	0.85±0.03 (150)
	Presynaptic area (um^2)	345.9±18.9 (46)	484.3±51.1 (34) (p=0.006 vs A)**	481.5±61.5 (14)	407.7±31.4 (23)
	Number of active zones	291.6±13.5 (31)	387.5±11.8 (18) (p<0.001 vs A)***	381.31±19.2 (16) (p=0.0048 vs D)**	284.4±22.7 (17)

Statistical significance was tested by Two-way ANOVA followed by Sidak's multiple comparisons test, **p*< 0.033 ** *p*< 0.002, *** *p*< 0.001

Supplementary Table 7: Electrophysiological measurements of indicated genotypes in adult HB9 motor neuron terminals at 29°C

**Genotypes (Aged day 35)**	***Evoked (EJC) Amplitude (nA)***	***Miniature (mEJC) Frequency (Hz)***	***Miniature (mEJC) Amplitude (nA)***
**(A) RNAi Control for Para^{KD}**<br>*UAS>luciferase^{RNAi} / HB9>Gal4, UAS>GFP,Tub>Gal80^{ts}*	0.94±0.22 (6)	0.79±0.26 (6)	0.16±0.006 (6)
**(B) Inhibition of EJC (Para^{KD})**<br>*UAS>GFP/+; HB9>Gal4,Tub>Gal80^{ts}/UAS>Para^{RNAi}*	0±0 (5)	0.71±0.09 (5)	0.18±0.01 (5)
**(C) Increase mEJC (mCPX^{HB})**<br>*UAS>Cpx^{RNAi}/UAS>GFP; HB9>Gal4,Tub>Gal80^{ts} / cpx^{SH1}, UAS>mCpx^{HB}*	n.a.	3.59±0.39 (7)	0.15±0.01 (7)

n.a. = not applicable

Supplementary Table 8: Primer sequences used in this study

Gene	Sequence	
$Kir_{2.1E}$	F: ATGGAGCAGAAGCTGATCAG	R: CTATATCTCCGATTCTCGCCTTAAGG
$dGluRIIA^{JA1}$ (To amplify genomic DNA)	F: CGCGACCGGCATTCCAC	R: TCATGAAGTAGGGCTCCTCG
