# Supplementary material for: Miniature neurotransmission is required to maintain Drosophila synaptic structures during ageing
Source: Nat Commun. 2021 Jul 20;12:4399. doi: 10.1038/s41467-021-24490-1 (PMC8292383; doi:10.1038/s41467-021-24490-1)
Supplement: Supplementary file 3 — Reporting summary [file 41467_2021_24490_MOESM3_ESM.pdf]

nature research

Corresponding author(s): Brian D. McCabe

Last updated by author(s): 2021/06/08

Reporting Summary

Nature Research wishes to improve the reproducibility of the work that we publish. This form provides structure for consistency and transparency in reporting. For further information on Nature Research policies, see our Editorial Policies and the Editorial Policy Checklist.

Statistics

For all statistical analyses, confirm that the following items are present in the figure legend, table legend, main text, or Methods section.

n/a	Confirmed	
☐	☒	The exact sample size (n) for each experimental group/condition, given as a discrete number and unit of measurement
☐	☒	A statement on whether measurements were taken from distinct samples or whether the same sample was measured repeatedly
☐	☒	The statistical test(s) used AND whether they are one- or two-sided<br>*Only common tests should be described solely by name; describe more complex techniques in the Methods section.*
☒	☐	A description of all covariates tested
☐	☒	A description of any assumptions or corrections, such as tests of normality and adjustment for multiple comparisons
☐	☒	A full description of the statistical parameters including central tendency (e.g. means) or other basic estimates (e.g. regression coefficient) AND variation (e.g. standard deviation) or associated estimates of uncertainty (e.g. confidence intervals)
☐	☒	For null hypothesis testing, the test statistic (e.g. F, t, r) with confidence intervals, effect sizes, degrees of freedom and P value noted<br>*Give P values as exact values whenever suitable.*
☒	☐	For Bayesian analysis, information on the choice of priors and Markov chain Monte Carlo settings
☒	☐	For hierarchical and complex designs, identification of the appropriate level for tests and full reporting of outcomes
☐	☒	Estimates of effect sizes (e.g. Cohen's d, Pearson's r), indicating how they were calculated

Our web collection on statistics for biologists contains articles on many of the points above.

Software and code

Policy information about availability of computer code

Data collection	Zeiss Zen 2 or 3, Mini Analysis 6.03 (synaptosoft), pClamp11, Imaris
Data analysis	Mini Analysis, pClamp 11, Image J, Adobe Photoshop 2021, GraphPad Prism 9, Imaris 5D image analysis (Custom macros publicly available at https://doi.org/10.5281/zenodo.4912667)

For manuscripts utilizing custom algorithms or software that are central to the research but not yet described in published literature, software must be made available to editors and reviewers. We strongly encourage code deposition in a community repository (e.g. GitHub). See the Nature Research guidelines for submitting code & software for further information.

Data

Policy information about availability of data

All manuscripts must include a data availability statement. This statement should provide the following information, where applicable:

- Accession codes, unique identifiers, or web links for publicly available datasets
- A list of figures that have associated raw data
- A description of any restrictions on data availability

Full data availability statement provided. Along with the Source data provided with manuscript, all original microscopy images and electrophysiology recordings are freely accessible at- https://doi.org/10.5281/zenodo.4906299

Field-specific reporting

Please select the one below that is the best fit for your research. If you are not sure, read the appropriate sections before making your selection.

- [x] Life sciences
- [ ] Behavioural & social sciences
- [ ] Ecological, evolutionary & environmental sciences

For a reference copy of the document with all sections, see nature.com/documents/nr-reporting-summary-flat.pdf

Life sciences study design

All studies must disclose on these points even when the disclosure is negative.

Sample size	Sample sized were predetermined based on previous studies in this field (at least 50 animals for behavioral experiments and at least 5 animals for confocal experiments). All sample sizes are indicated in each figure legend and in the supplementary tables.
Data exclusions	No data was excluded from our analyses.
Replication	All data presented are representative of at least three independent experiments as indicated in the method part of statistics, and replications were successful.
Randomization	All Neuromuscular junction (NMJ) synapses and flies analyzed in this study were randomly selected.
Blinding	Samples were blinded during preparation and analysis.

Reporting for specific materials, systems and methods

We require information from authors about some types of materials, experimental systems and methods used in many studies. Here, indicate whether each material, system or method listed is relevant to your study. If you are not sure if a list item applies to your research, read the appropriate section before selecting a response.

Materials & experimental systems

n/a	Involved in the study	
[ ]	[x]	Antibodies
[x]	[ ]	Eukaryotic cell lines
[x]	[ ]	Palaeontology and archaeology
[ ]	[x]	Animals and other organisms
[x]	[ ]	Human research participants
[x]	[ ]	Clinical data
[x]	[ ]	Dual use research of concern

Methods

n/a	Involved in the study	
[x]	[ ]	ChIP-seq
[x]	[ ]	Flow cytometry
[x]	[ ]	MRI-based neuroimaging

Antibodies

Antibodies used	Chicken or Rabbit anti-GFP (1:1000; AbCam), Rabbit anti-RFP (1:500; Clonetech), Mouse anti-DLG (1:100; Developmental Studies Hybridoma Bank [DSHB]), Mouse anti-Brp (Nc82, 1:100; DSHB), Rabbit anti GluRIIC (1:100, Gift from Stephan Sigrist)71, Mouse anti-GluRIIA (1:20; DSHB), Guinea Pig anti-GluRIID (1:500, this study), Mouse anti-VGLUT(1:100, this study), Chicken anti-VGLUT (1:100, this study), Rabbit anti-VGLUT, (1:1000; Gift from Hermann Aberle), Mouse anti-VMAT(1:100, this study), Rabbit anti-Synaptotagmin (1:1000; Gift from Troy Littleton), Rabbit anti-Complexin (1:1000, Gift from Troy Littleton), Mouse anti-22C10 (1:100, DSHB), Mouse anti-tubulin (1:100, DSHB), Rabbit anti-Myc (1:500, Cell Signaling), Guinea Pig anti-V100 (1:1000, Gift from Robin Heisinger), goat anti-chicken (Alexa-488, 555 or 647; 1:400), goat anti-mouse (Alexa-Cy3 or 647; 1:400), goat anti-rabbit (Alexa-488, 546 or 647; 1:400) goat anti-Guinea Pig (555, 1:400), Alexa Flour 488-HRP (1:400; Jackson ImmunoResearch)
Validation	Mouse anti-VGLUT(1:100), Chicken anti-VGLUT (1:100), Mouse anti-VMAT (1:100), Guinea Pig anti-GluRIID (1:500) antibodies were generated and validated in this study. All other antibodies were validated in other prior studies to which references are provided.

Animals and other organisms

Policy information about studies involving animals; ARRIVE guidelines recommended for reporting animal research

Laboratory animals	All Drosophila melanogaster strain information is provided in the Methods under the section 'Drosophila stocks'. Only male flies were used in this study.
Wild animals	The study did not involve wild animals
Field-collected samples	No field-collected samples were used

Ethics oversight	This study did not require any ethical approval

Note that full information on the approval of the study protocol must also be provided in the manuscript.
